# A Study on Minimizing Welding Deformation of Joints for the Sealing of Emission After-Treatment Structure

**DOI:** 10.3390/ma14226982

**Published:** 2021-11-18

**Authors:** Sungwook Kang, Wangho Yun, Hwanjin Kim, Jaewoong Kim, Changwook Ji, Kwangjin Lee, Jaehwang Kim, Hong-Lae Jang, Kwangsan Chun

**Affiliations:** 1 Precision Mechanical Process and Control R&D Group, Korea Institute of Industrial Technology, Jinju-si 52845, Korea; swkang@kitech.re.kr (S.K.); whyun@kitech.re.kr (W.Y.); 2 Electromagnetic Wave Fusion Research Department, Gyeongbuk Research Institute of Vehicle Embedded Technology, Yeongcheon-si 38822, Korea; hjkim@givet.re.kr; 3 Automotive Materials & Components R&D Group, Korea Institute of Industrial Technology, Gwangju 61012, Korea; kjw0607@kitech.re.kr; 4 Advanced Forming Process R&D Group, Korea Institute of Industrial Technology, Ulsan-si 44413, Korea; cwji@kitech.re.kr; 5 Carbon & Light Materials Application R&D Group, Korea Institute of Industrial Technology, Jeonju-si 54853, Korea; kjlee@kitech.re.kr (K.L.); raykim@kitech.re.kr (J.K.); 6 School of Mechanical Engineering, Changwon National University, Changwon-si 51140, Korea; 7 Industrial Application R&D Group, Welding Engineering R&D Department, Daewoo Shipbuilding & Marine Engineering Co., Ltd., Geoje-si 53302, Korea

**Keywords:** thermal elasto-plastic analysis, welding sequence, muffler structure, finite element method, welding deformation

## Abstract

As the environmental pollution issue has recently become significant, environmental regulations in Europe and the United States are being strengthened. Thus, there is a demand for the quality improvement of emission after-treatment systems to satisfy the strengthened environmental regulations. Reducing the amount of welding heat distortion by optimization of the welding order of each part could be a solution for quality improvement since the emission after-treatment system consists of many parts and each assembly is produced by welding individual ones. In this research, a method to derive a welding sequence that effectively minimizes welding deformation was proposed. A two-stage simulation was performed to obtain the optimal welding sequence. In the first stage, the welding sequence was derived by analyzing the number of welding groups in each assembly of a structure. The derived welding sequence was verified by performing a thermal elasto-plastic analysis and comparing it with the experimental results.

## 1. Introduction

There are residual stresses and deformation on a welding part due to local heating and cooling that occurs during the welding process. In fusion welding, fusion occurs when a molten part is created locally due to intensive heat input and the surrounding area near the fusion part goes through heating and cooling processes while forming a non-uniform temperature distribution. These residual stresses and deformations cause cracks, shape and dimensional errors when a welded structure is produced. In general, a metal expands and softens at the same time as it is heated, therefore reducing the deformation resistance. At extremely high temperatures, there is almost no resistance, so the stress at high temperatures is not very large. However, significant shrinkage and tensile residual stress are always present in a welding part since the shrinkage stress generated during cooling increases with the temperature drop. This thermal stress creates thermal deformation and the deformation that remains permanently after the welding process is called welding deformation. The welding deformation occurring in a welded structure changes the dimensions of the structure or makes the assembly process after the welding process difficult, so additional after-treatment process such as correction is required.

This study focuses on the structure of an emission after-treatment system that can comply with the strengthened environmental regulations. The device can be applied to off-road vehicles such as construction machinery, agricultural machinery, and specialty vehicles and is designed to comply with the Euro 6 emission regulation policy. The Euro 6 for vehicles in Europe, the strictest emission regulation, requires the application of a diesel particle filter (DPF) that filters out particulates in an exhaust gas. The DPF is a type of exhaust gas after-treatment device that physically collects and burns particulate matters in the exhaust gas of a diesel engine. In the United States, the on-board diagnostics (OBD) were enacted for automobile emission control laws in accordance with the regulations for an automobile self-diagnosis device. In Europe and the United States, these regulations have been enacted to reduce the emissions of nitrogen oxides (NOx), carbon monoxide (CO), hydrocarbons (HC), and particulate matter (PM). As the environmental pollution issue has recently become significant, there is a demand for the quality improvement of an emission after-treatment system, which is an assembly of a number of parts such as catalysts and sensor mixers, as well as advanced catalytic technology due to the reinforcement of emission gas regulations such as elevated related regulations, introduction of defect check inspection system, and extension of exhaust gas warranty period, etc. The emission after-treatment system consists of many parts (chamber, mixer, plate, cover, boss, case, tube, bracket, etc.). These parts configure a diesel oxidation catalyst (DOC) assembly, DPF assembly, and outlet assembly and these assemblies configure an emission after-treatment device. Since a catalyst is added into the emission after-treatment device, the catalyst cannot be exchanged later if the assemblies are welded, so the individual assemblies are clamped. Since each assembly is produced by welding individual parts, there is an issue that leakage occurs in a fastening joint during final clamping due to welding heat deformation in each part. To solve this problem takes a lot of time and cost for additional post-processing. Since each assembly consists of 3 to 10 welding parts, accumulated tolerances during welding deteriorates the quality of the assembly. Reducing the amount of welding heat distortion by optimizing the welding order of each part could be a fundamental solution.

Numerous numerical methods have been proposed to predict the amount of welding deformation [1,2]. Thermal elasto-plastic analysis is used for simulation purposes to accurately calculate welding deformation, but it takes a long time to perform the analysis [1,2,3]. Deng et al. [4] performed a 3-D thermal elasto-plastic finite element analysis to determine the welding deformation of fillet-welded joints. In addition, experiments were conducted to investigate the characteristics of welding deformation and these were compared with the results of numerical analysis. The FE simulation involved two steps. First, a heat conduction analysis was performed, and then a mechanical analysis according to the thermal load was performed from the temperature history obtained through the heat conduction analysis. Many studies have been undertaken to improve the solution efficiency of thermal elasto-plastic finite element analysis with regard to welding phenomena. Reducing the number of nodes using 3D/shell elements reduces the simulation time [5,6,7]. Murakawa et al. [8] proposed an iterative substructure method (ISM) to reduce the simulation time. When ISM is applied to welding deformation problems, the model is divided into a strong nonlinear region around the weld pool and a weak non-linear region [9,10]. Recently, parallel computation methods using graphical processing units (GPU) have been utilized to accelerate computation speed for welding deformation prediction [11,12,13,14].

On the other hand, the simplified method can shorten the analysis time, but the accuracy is lower than that of thermal elasto-plastic analysis [15,16,17,18,19]. Lee et al. [20] developed an FE-modeling method for efficient welding angular distortion prediction. The force matrix in the FE formulation is derived explicitly to transform the scalar input variable considering the mesh size. Rong et al. [21] performed laser butt welding experiments to observe the characteristics of a keyhole and weld pool. The geometries of the keyhole and weld pool were extracted by high speed imaging and image processing. For the prediction of weld size, a double cylindrical heat source model considering the keyhole angle and diameter was derived and validated using the measured weld pool geometries.

When optimizing the welding sequence and accurately predicting the final welding deformation, thermal elasto-plastic analysis has a limitation in its application due to the time taken to perform the analysis. Gannon et al. [22] presented the effect of welding sequence on the flat-bar stiffened plates. Sequential coupled thermal and structural finite element analyses were performed to investigate the residual stress and welding distortion. To model the addition of weld metal to a workpiece, an element birth and death technique was utilized. The developed FE model was verified based on the experimental results reported by Deng et al., [4] and the residual stresses and distortions of flat-bar stiffened plates considering four different welding sequences were analyzed with the FE model. Tabar et al. [23] and Heung et al. [24] used a genetic algorithm to optimize the spot-welding sequence. L Romanin et al. [25] divided a large welded structure into different local welded structures, calculated them independently using the equivalent load method, and calculated the welding deformation by combining them. Kadivar et al. [26] compared simulation and analysis results using thermal elasto-plastic analysis for a two-dimensional finite element model. A genetic algorithm was used to optimize the welding sequence. Kang et al. [27] optimized the welding sequence for block assemblies in the shipbuilding industry. In particular, they focused on productivity by considering workability. Romero-Hdz et al. [28] introduced artificial intelligent techniques to optimize the welding sequence. However, there was no experimental verification and the factors required to calculate the amount of welding deformation such as residual stress and temperature were not considered. Ha [29] derived an optimal welding sequence using the elasto-plastic strain-boundary method. In the study, the calculation time was shortened by deriving the welding sequence using a simplified method, but there is a limitation because only qualitative results can be predicted.

In this study, a method to derive a welding sequence that effectively minimizes welding deformation was proposed. The welding sequence was derived by analyzing the number of welding groups in each assembly of a structure. Using the derived welding sequence, thermal elasto-plastic analysis was performed to calculate the amount of welding deformation of an actual structure and it was compared with the experimental results. The proposed method was verified by performing a simulation to derive the sequence of minimizing the welding heat deformation amount of each part for the emission after-treatment system structure and comparing it with a case where the actual optimal process was applied.

## 2. Simulation to Minimize Welding Deformation Using Finite Element Method

### 2.1. Muffler Structure Welding Experiments Condition and Welding Section

**Figure 1:**
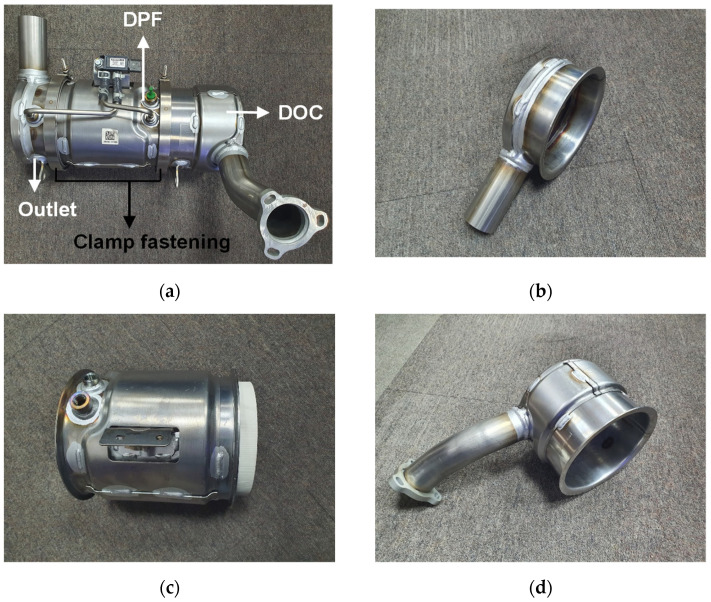
Actual muffler structure and each assembly: (**a**) entire muffler structure; (**b**) outlet assembly; (**c**) diesel particle filter (DPF) assembly; (**d**) diesel oxidation catalyst (DOC) assembly.

The muffler structure, the subject of this study, consists of a DOC, DPF, and outlet assembly as shown in Figure 1. As shown in Figure 1, each assembly is connected by clamping. This has a great advantage compared to welding in terms of internal filter replacement and maintenance. However, an issue may arise in terms of product performance if airtightness is not secured at the fastening part.

The outlet assembly used the same 2.0 mm thickness. For the DPF assembly, a thickness of 2.0 mm and a thickness of 12.0 mm were used. The DOC assembly used two types of welding combination. The first has a thickness of 2.0 mm and a thickness of 1.0 mm. The second has a thickness of 2.0 mm and a thickness of 4.0 mm. All structures are constructed of stainless steel 436.

**Figure 2:**
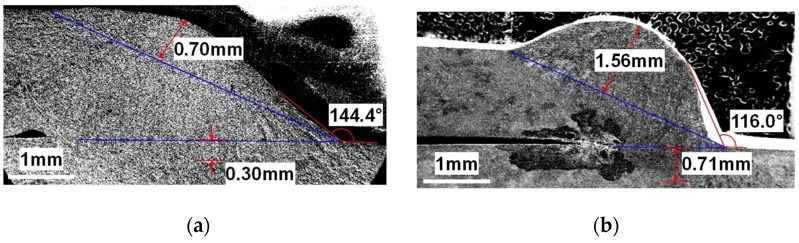

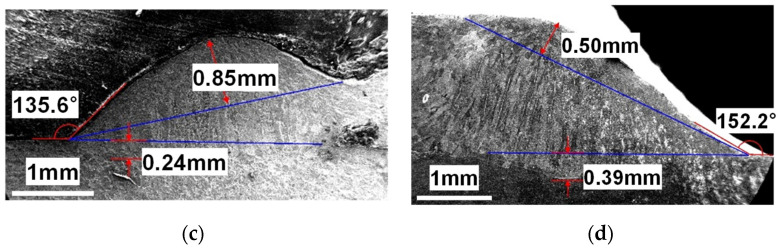
Bead shape for each assembly: (**a**) outlet assembly (2.0 mm–2.0 mm combination); (**b**) DPF assembly (2.0 mm–12.0 mm combination); (**c**) DOC assembly (2.0 mm–1.0 mm combination); (**d**) DOC assembly (2.0 mm–4.0 mm combination).

After welding for each assembly, the shape of the bead was measured as shown in Figure 2. The measured shape of the bead was reflected in the bead modeling when the simulation was performed.

SF-436 with a diameter of 1.2 mm was used as a filler material for welding the muffler structure. The welding equipment used in this study was Fronius’ TPS4000, and automatic welding was performed using a six-axis robot. The contact tip to working distance (CTWD) was fixed at 15 mm and the torch angle was fixed at 55°. As a protective gas, 100% argon gas was continuously supplied at 20 L/min. The welding current and welding speed were set to 150 A and 10 mm/s, respectively.

### 2.2. Methodology

**Figure 3:**
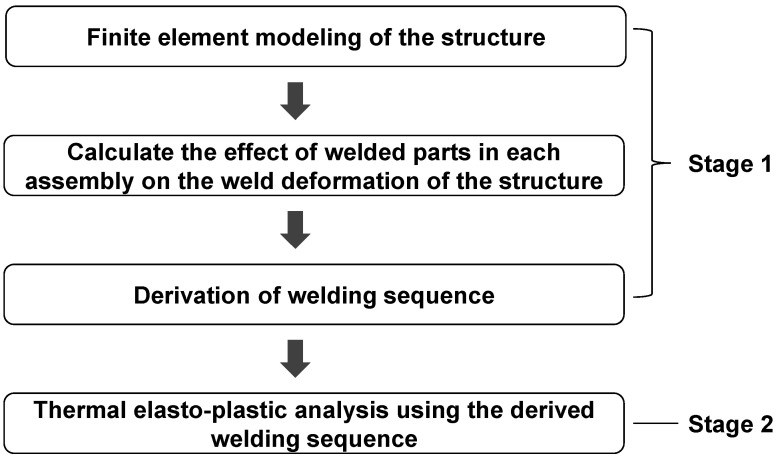
Finite element (FE) analysis flow chart for minimizing welding deformation.

In this study, two-stage simulation was performed. In stage 1, the sequence of minimizing the amount of welding deformation for each assembly was derived. In stage 2, based on the sequence derived in stage 1, the thermal elasto-plastic analysis was performed to calculate the amount of welding deformation of a structure. The flow chart to calculate the final welding deformation is shown in Figure 3.

### 2.3. Stage 1: Deriving the Welding Sequence to Minimize Thermal Deformation of Joints

**Figure 4:**
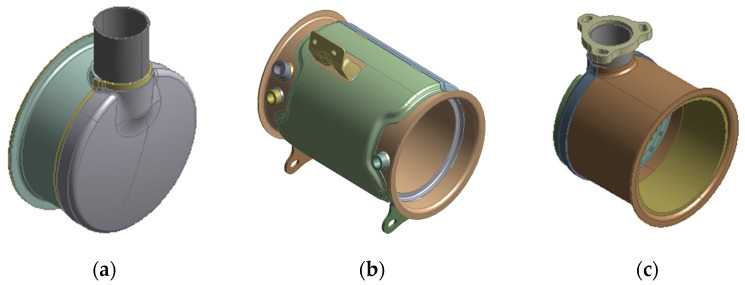
FE model of each assembly of the muffler structure: (**a**) outlet assembly; (**b**) DPF assembly; (**c**) DOC assembly.

Each assembly went through finite element modeling for simulation as shown in Figure 4. To increase the simulation efficiency, a part that does not affect the deformation of a structure was simplified or removed to make the final FE model.

**Figure 5:**
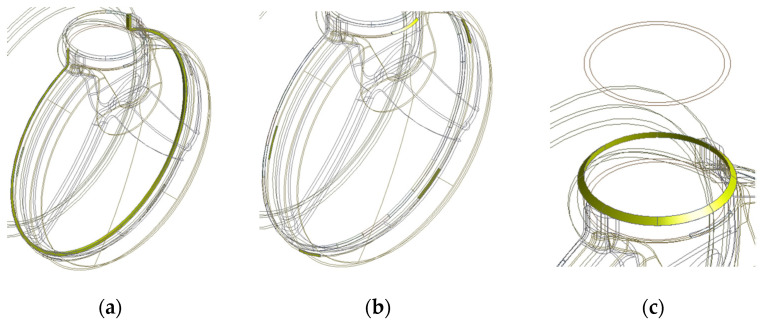
Welding parts of outlet assembly: (**a**) Part 1; (**b**) Part 2; (**c**) Part 3.

**Figure 6:**
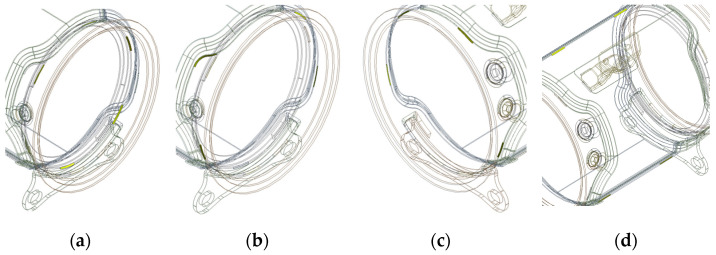

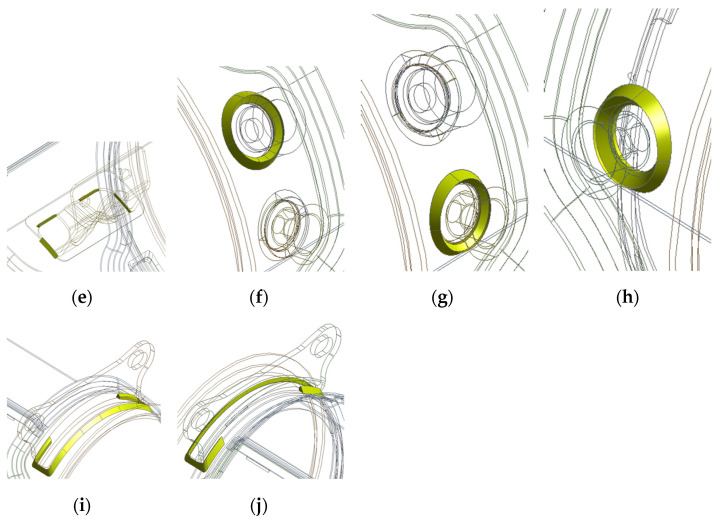
Welding parts of DPF assembly: (**a**) Part 1; (**b**) Part 2; (**c**) Part 3; (**d**) Part 4; (**e**) Part 5; (**f**) Part 6; (**g**) Part 7; (**h**) Part 8; (**i**) Part 9; (**j**) Part 10.

**Figure 7:**
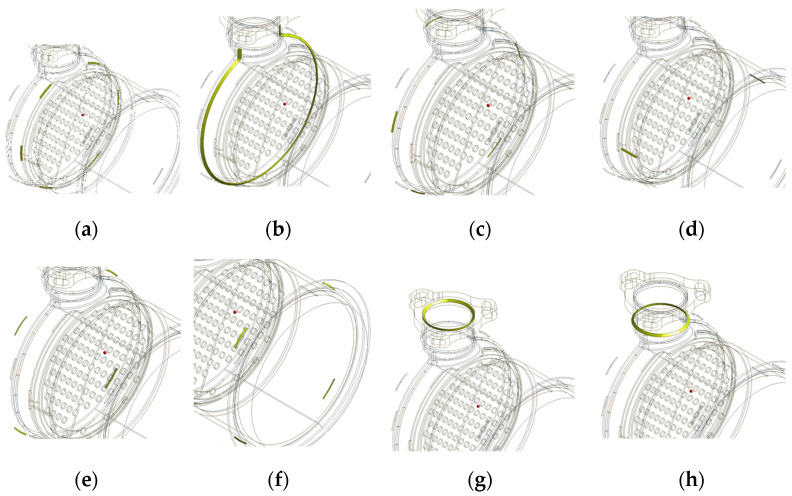
Welding parts of DOC assembly: (**a**) Part 1; (**b**) Part 2; (**c**) Part 3; (**d**) Part 4; (**e**) Part 5; (**f**) Part 6; (**g**) Part 7; (**h**) Part 8.

The parts are welded to form each assembly structure. In Figure 4, outlet assembly, DPF assembly, and DOC assembly have 3, 10, and 8 welding parts, respectively. In the case of a DPF assembly, there are 10 welding parts, so the total possible welding sequence is 10!, i.e., 3,628,800. This is an impossible figure even for an experiment and simulation. Therefore, in this study, to solve this problem, the effect of each welding part on the welding deformation of a structure was first calculated. For the objective, the welding parts of each assembly are as shown in Figure 5, Figure 6 and Figure 7. The yellow solid part in Figure 5, Figure 6 and Figure 7 shows a bead that is the joint of each welding part.

A simulation was performed as follows to identify the effect of each welding part on the welding deformation of a structure. For example, to calculate the effect of Part 6 of the DPF assembly on the welding deformation of a structure, thermal elasto-plastic analysis was performed on Part 6 to calculate the welding deformation on both joints of the DPF assembly after making an FE model that excludes Part 6 of the DPF assembly.

### 2.4. Stage 1: Calculating the Effect of Welding Parts on Welding Deformation Using Thermoplastic Analysis

As mentioned earlier, the total number of simulation cases to calculate the effect of welding parts of each assembly on welding deformation is as many as the number of corresponding welding parts since the number of welding parts of outlet assembly, DPF assembly, and DOC assembly are 3, 10, and 8, respectively. In other words, the total number of welding cases of DPF was 10!, but the number of simulations was drastically reduced to 10.

To perform the thermal elasto-plastic analysis, the temperature-dependent of material properties of stainless steel 436, i.e., the material of a muffler structure, was used [30]. The chemical composition of stainless steel 436 is summarized in Table 1.

This simulation used Ansys Workbench 2021 R1 to implement a welding process environment similar to the actual environment. The finite element model was meshed using three-dimensional solid elements (SOLID186 and SOLID187 in Ansys). The transient thermal elasto-plastic analysis was performed at the actual welding speed of 10 mm/s and the time step size was set to 2 s. A specimen test was performed to calculate the heat source during welding and the heat source in the final welding part was estimated by measuring the temperature data using a thermocouple. In the heat transfer analysis, the natural convection condition was applied to the surface of a structure exposed to the air. The convective heat transfer coefficient under the natural convection condition was 20 W/m^2^·K and the convective heat transfer coefficient at the surface in contact with a jig was 200 W/m^2^·K, and the analysis was performed in an environment similar to the actual welding process [31]. In addition, to enable the creation of a moving heat source and a bead during welding, a welding bead element was configured to be created according to each time step. For this, experiments were performed according to the welding conditions in Section 2.1 to reflect the bead shape of each assembly in the simulation model. In addition, in order to realize beads that are generated according to the time step during simulation, the element alive function (EALIVE) in Ansys was used. Similarly, the parts of each assembly were set to generate a finite element model according to the time step and welding sequence.

After simulation on the welding parts of each assembly using these conditions, the welding deformation at the joint was calculated and shown in Table 2, Table 3 and Table 4. In each assembly, the welding deformation ratio of each part was calculated after the result of a part with the smallest welding deformation was set to 1. For example, if the Part 1 of Table 1 outlet assembly affects deformation at the joint by 1, it means that Part 2 and Part 3 affect the welding deformation by 1.11 and 5.98 times. Therefore, Part 3 of the outlet assembly, which has the greatest influence on welding deformation at the joint of a final structure, should be welded last. This is because the rigidity of a structure increases and welding deformation can be suppressed when other structures are welded first. For the rest of the assemblies, the welding deformation of each welding part was calculated and converted into a ratio. After that, the sequence was determined to enable a welding part that causes a large amount of welding deformation to be welded later. However, since the result calculated from the thermal elasto-plastic analysis does not reflect the actual process, the sequence may be changed considering the actual process. This is because there may be a situation where the inside of a structure cannot be welded in the process if the outer part of a structure is welded first. Therefore, the order was finally determined by considering the process and simulation results.

In Table 2, in the case of the DPF, the welding sequence was determined using the average value at two positions because the fastening part exists on both sides.

### 2.5. Stage 2: Calculating Welding Deformation at Joints by Considering Welding Sequence

**Figure 8:**
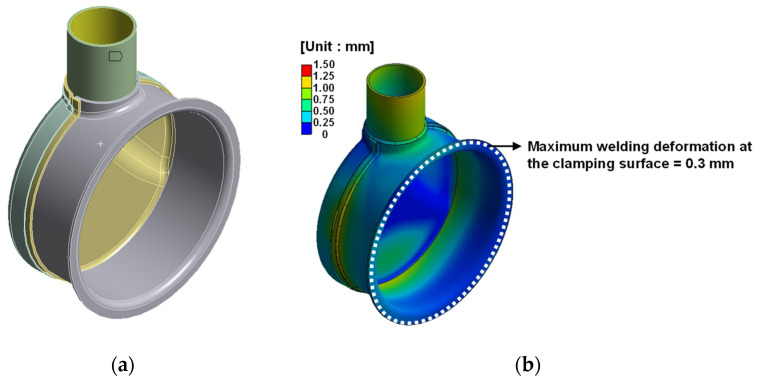
Welding deformation of outlet assembly: (**a**) FE model of outlet assembly; (**b**) welding deformation result.

**Figure 9:**
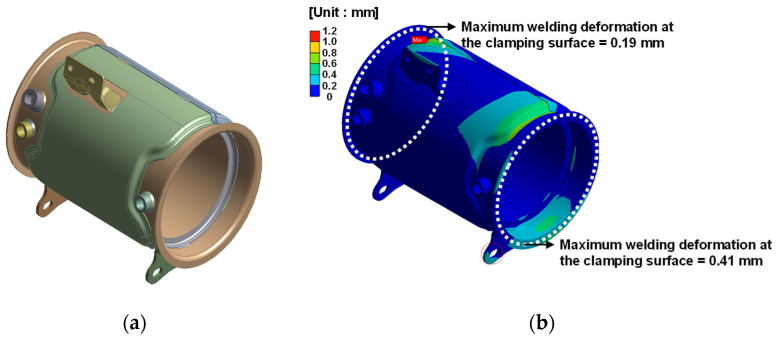
Welding deformation of DPF assembly: (**a**) FE model of DPF assembly; (**b**) welding deformation result.

**Figure 10:**
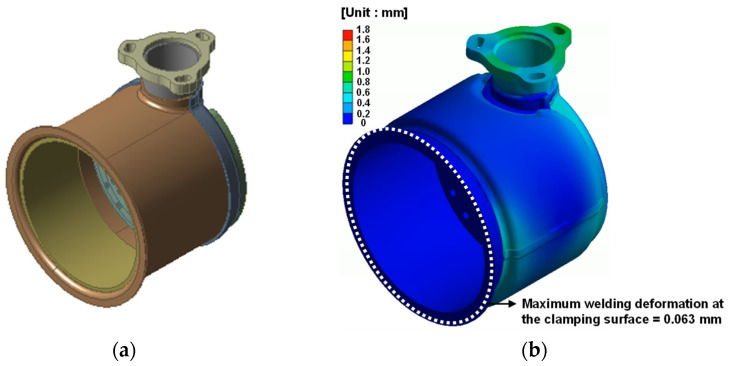
Welding deformation of DOC assembly: (**a**) FE model of DOC assembly; (**b**) welding deformation result.

By considering the process and simulation results, the welding sequence that can minimize the welding deformation at the clamp joint was derived for each assembly. A thermal elasto-plastic analysis was performed for each assembly using the derived welding sequence. Welding deformation at the joint was calculated using the same welding conditions as in Section 2.4. Figure 8, Figure 9 and Figure 10 shows the welding deformation at the joint of each assembly.

From the thermal elasto-plastic analysis, the welding deformation on the fastening surface of each assembly was found to be less than a maximum 0.41 mm.

## 3. Muffler Structure Welding Experiment Considering the Optimal Sequence

The welding sequence was derived from Section 2.4 and the welding deformation at the fastening surface of each assembly was calculated using the thermal elasto-plastic analysis in Section 2.5. To verify the simulation results, the muffler structure was produced according to the welding sequence derived from Section 2.4 and the welding deformation was measured.

**Figure 11:**
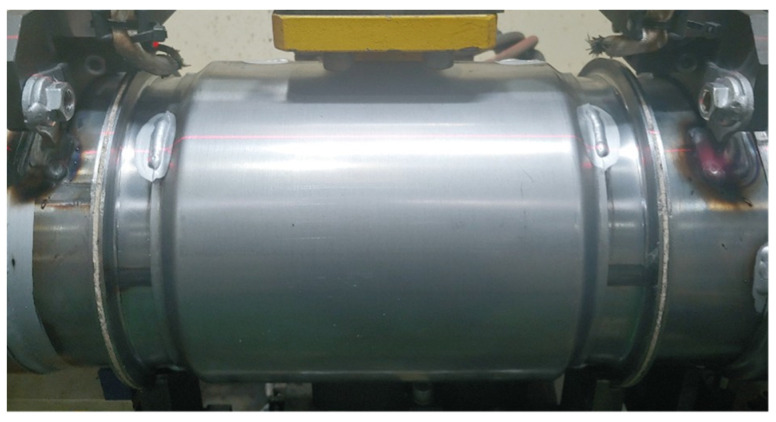
Welding of the muffler structure.

Figure 11 shows the welding of the muffler structure.

**Figure 12:**
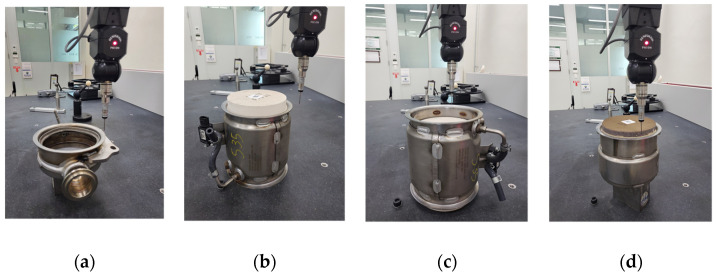
Measurement of welding deformation at clamping surface of muffler structure using a 3D scanner: (**a**) outlet assembly; (**b**) DPF assembly–outlet assembly; (**c**) DPF assembly–DOC assembly; (**d**) DOC assembly.

Surveyor model DS-4060 (Laser Design Inc., Minneapolis, MN, USA) 3D scanner was used to measure the amount of welding deformation at the joint of a welded assembly. Figure 12 shows the measurement of welding deformation in each assembly. In the case of the DPF assembly, it is located in the middle, so the outlet assembly and DOC assembly are fastened at both sides. Figure 12b is the surface connected to the outlet assembly and Figure 12c is the surface connected to the DOC assembly.

The experimental results and elasto-plastic analysis results for the welding deformation of the final muffler structure are summarized in Table 5.

When comparing the experimental and simulation results, these show that the maximum welding deformation error at the joint is 13.89% or less. The allowable deformation of the clamping surface is under 0.5 mm. This is a value that does not cause leakage problems during the airtightness test after the final assembly of the muffler structure.

## 4. Conclusions

In the case of a structure with many welding parts, it is impossible to calculate all the parts through experiments and simulations because the number of cases for welding sequence increases exponentially. Therefore, in this study, a method to derive a welding sequence that effectively minimizes welding deformation was proposed. First, to derive the sequence of minimizing the welding deformation in each assembly joint, the effect of welding parts on the welding deformation of a structure was calculated, respectively. Based on the calculated results, the sequence was derived so that the part that has the greatest influence on welding deformation was welded last. Since the rigidity of a structure increases when other parts are welded first, it is advantageous in terms of welding deformation of an entire structure when the parts that significantly affect the welding deformation are welded later.

In this study, the welding sequence showing the minimum welding deformation at the joint of each assembly was derived for an assembly in which there are 3, 10, and 8 welding parts, respectively. Based on the results, the amount of welding deformation was calculated using thermal elasto-plastic analysis. To verify the simulation results, an experiment was performed using the same process as the simulation and then the welding deformation was measured. Through this process, it was verified that the proposed analysis method is appropriate, and it is expected to be of great help when determining the welding sequence of structures with many weld lines.

## Figures and Tables

**Table 1:** The chemical composition of stainless steel 436.

Component	Weight Percent (wt%)
Carbon	0.12
Chromium	16.0–18.0
Molybdenum	0.75–1.25
Manganese	1.00
Silicon	1.00
Phosphorus	0.04
Sulfur	0.03

**Table 2:** Welding sequence for outlet assembly.

	Part 1	Part 2	Part 3
Result	1.00	1.11	5.98
Welding sequence by simulation result	1	2	3
Welding sequence considering the work process and simulation results	1	2	3

**Table 3:** Welding sequence for DPF assembly.

	Part 1	Part 2	Part 3	Part 4	Part 5
Result	4.70	15.43	1.85	1.00	3.33
Welding sequence by simulation result	6	8	2	1	4
Welding sequence considering the work process and simulation results	6	8	2	1	4
	**Part 6**	**Part 7**	**Part 8**	**Part 9**	**Part 10**
Result	7.03	3.39	3.21	23.29	16.73
Welding sequence by simulation result	7	5	3	10	9
Welding sequence considering the work process and simulation results	7	5	3	10	9

**Table 4:** Welding sequence for DOC assembly.

	Part 1	Part 2	Part 3	Part 4
Result	2.98	20.39	1.00	7.38
Welding sequence by simulation result	2	7	1	5
Welding sequence considering the work process and simulation results	1	2	4	5
	**Part 5**	**Part 6**	**Part 7**	**Part 8**
Result	3.23	39.41	4.97	16.27
Welding sequence by simulation result	3	8	4	6
Welding sequence considering the work process and simulation results	3	8	7	6

**Table 5:** Comparison of experimental and simulation results for welding deformation of the clamping surface.

	Outlet Assembly	DPF Assembly–Outlet Assembly	DPF Assembly–DOC Assembly	DOC Assembly
Experimental [mm]	0.28	0.36	0.17	0.071
Simulation [mm]	0.30	0.41	0.19	0.063
Agreement [%]	7.14	13.89	11.76	11.27

## Data Availability

Not applicable.

## References

[1] Kang S., Kim J., Kim D., Jang Y., Cho J. Thermal Elasto-Plastic Analysis of Welding Sequence for Least Distortion of Overlay Welded Structure. Proceedings of the Volume 3: Structures, Safety, and Reliability, American Society of Mechanical Engineers.

[2] Rong Y., Xu J., Huang Y., Zhang G. (2017). Review on finite element analysis of welding deformation and residual stress. Sci. Technol. Weld. Join..

[3] Narang H.K., Mahapatra M.M., Jha P.K., Sridhar P., Biswas P. (2018). Experimental and Numerical Study on Effect of Weld Rein-forcement on Angular Distortion of SAW Square Butt Welded Plates. J. Weld. Join..

[4] Deng D., Liang W., Murakawa H. (2007). Determination of welding deformation in fillet-welded joint by means of numerical simu-lation and comparison with experimental results. J. Mater. Process. Technol..

[5] Rong Y., Zhang G., Huang Y. (2017). Study on deformation and residual stress of laser welding 316L T-joint using 3D/shell finite element analysis and experiment verification. Int. J. Adv. Manuf. Technol..

[6] Rong Y., Zhang G., Huang Y. (2016). Study of Welding Distortion and Residual Stress Considering Nonlinear Yield Stress Curves and Multi-constraint Equations. J. Mater. Eng. Perform..

[7] Kim J.W., Jang B.S., Kang S.W. (2014). A study on an efficient prediction of welding deformation for T-joint laser welding of sandwich panel Part II: Proposal of a method to use shell element model. Int. J. Nav. Arch. Ocean Eng..

[8] Murakawa H., Oda I., Itoh S., Serizwa H., Shibahara M., Nishikawa H. (2004). Iterative substructure method for fast FEM analysis of mechanical problems in welding. Prepr. Natl. Meet. JWS.

[9] Huang H., Ma N., Hashimoto T., Murakawa H. (2015). Welding deformation and residual stresses in arc welded lap joints by modified itera-tive analysis. Sci. Technol. Weld. Join..

[10] Murakawa H., Ma N., Huang H. (2015). Iterative substructure method employing concept of inherent strain for large-scale welding problems. Weld. World.

[11] Ikushima K., Shibahara M. (2014). Prediction of residual stresses in multi-pass welded joint using Idealized Explicit FEM accelerated by a GPU. Comput. Mater. Sci..

[12] Ikushima K., Itoh S., Shibahara M. (2015). Development of idealized explicit FEM using GPU parallelization and its application to large-scale analysis of residual stress of multi-pass welded pipe joint. Weld. World.

[13] Ma N. (2016). An accelerated explicit method with GPU parallel computing for thermal stress and welding deformation of large structure models. Int. J. Adv. Manuf. Technol..

[14] Ma N. (2016). An accelerated explicit method and GPU parallel computing for thermal stress and welding deformation of automo-tive parts. Int. J. Appl. Mech..

[15] Kim Y., Kim J., Kang S. (2019). A Study on Welding Deformation Prediction for Ship Blocks Using the Equivalent Strain Method Based on Inherent Strain. Appl. Sci..

[16] Kang S., Kim J., Jang Y., Lee K. (2019). Welding Deformation Analysis, Using an Inherent Strain Method for Friction Stir Welded Electric Vehicle Aluminum Battery Housing, Considering Productivity. Appl. Sci..

[17] Wu C., Wang C., Kim J.-W. (2021). Welding Distortion Prediction for Multi-Seam Welded Pipe Structures using Equivalent Thermal Strain Method. J. Weld. Join..

[18] Kim T.-J., Jang B.-S., Kang S.-W. (2015). Welding deformation analysis based on improved equivalent strain method considering the effect of temperature gradients. Int. J. Nav. Archit. Ocean Eng..

[19] Kim T.J., Jang B.S., Kang S.W. (2015). Welding deformation analysis based on improved equivalent strain method to cover ex-ternal constraint during cooling stage. Int. J. Nav. Archit. Ocean Eng..

[20] Lee J., Seo H., Chung H. (2018). Efficient welding distortion analysis method for large welded structures. J. Mater. Process. Technol..

[21] Rong Y., Wang L., Wu R., Xu J. (2022). Visualization and simulation of 1700MS sheet laser welding based on three-dimensional geometries of weld pool and keyhole. Int. J. Therm. Sci..

[22] Gannon L., Liu Y., Pegg N., Smith M. (2010). Effect of welding sequence on residual stress and distortion in flat-bar stiffened plates. Mar. Struct..

[23] Tabar R.S., Wärmefjord K., Söderberg R., Lindkvist L. (2019). A novel rule-based method for individualized spot welding se-quence optimization with respect to geometrical quality. J. Manuf. Sci. Eng..

[24] Huang M.-W., Hsieh C.C., Arora J.S. (1997). A genetic algorithm for sequencing type problems in engineering design. Int. J. Numer. Methods Eng..

[25] Romanin L., Ferro P., Berto F. (2021). A simplified non-linear numerical method for the assessment of welding induced defor-mations. Mar. Struct..

[26] Kadivar M.H., Jafarpur K., Baradaran G.H. (2000). Optimizing welding sequence with genetic algorithm. Comput. Mech..

[27] Kang M., Seo J., Chung H. (2018). Ship block assembly sequence planning considering productivity and welding deformation. Int. J. Nav. Arch. Ocean Eng..

[28] Romero-Hdz J., Saha B.N., Tstutsumi S., Fincato R. (2020). Incorporating domain knowledge into reinforcement learning to ex-pedite welding sequence optimization. Eng. Appl. Artif. Intell..

[29] Ha Y. (2013). Analytical Methodology Obtaining an Optimal Welding Sequence for Least Distortion of Welded Structure. J. Weld. Join..

[30] Lee S., Kim E., Park J., Choi J. (2017). MIG Welding Optimization of Muffler Manufacture by Microstructural Change and Ther-mal Deformation Analysis. J. Weld. Join..

[31] Kim H., Lee K., Kim J., Lee C., Jung Y., Kang S. (2020). A Study on the Friction Stir Welding Experiment and Simulation of the Fillet Joint of Extruded Aluminum Material of Electric Vehicle Frame. Appl. Sci..

